# Comparison of X-ray and CT Images of COVID-19 Caused by the Wild-Type and Alpha-Variant SARS-CoV-2

**DOI:** 10.7759/cureus.76493

**Published:** 2024-12-27

**Authors:** Chika Tsuchida, Ippei Sakamaki, Norichika Hashimoto, Toshiko Iwasaki, Yoshitomo Saiki, Yuzuru Takeuchi, Shinichi Katsuo, Hiromichi Iwasaki

**Affiliations:** 1 Department of Radiology, Fukui General Hospital, Fukui, JPN; 2 Department of Infectious Diseases, University of Fukui, Fukui, JPN; 3 Department of Neurosurgery, Fukui General Hospital, Fukui, JPN; 4 Division of Physical Therapy, Department of Rehabilitation, Fukui Health Science University, Fukui, JPN; 5 Department of Obstetrics and Gynecology, Fukui General Hospital, Fukui, JPN; 6 Department of Orthopedics, Fukui General Hospital, Fukui, JPN; 7 Division of Infection Control and Prevention, University of Fukui Hospital, Fukui, JPN

**Keywords:** alpha variant, computed tomography (ct), coronavirus disease 2019 (covid-19), pneumonia, wild-type

## Abstract

Introduction

This study aimed to determine the characteristics of coronavirus disease 2019 (COVID-19) pneumonia caused by the wild type and the alpha variant in patients. This study included patients with COVID-19 admitted to Fukui General Hospital between October 31, 2020, and April 30, 2021.

Methods

Pneumonia occurrence rate, chest X-ray, and computed tomography (CT) findings were evaluated by two radiologists. The time since the onset and presence of pneumonia were also investigated.

Results

Out of 128 patients, 43 had pneumonia. The pneumonia detection rates using chest radiography were 15.6% (20/128) and 33.6% (43/128) using CT (p = 0.0008). Of the pneumonia cases detected by CT, 32.0% (8/25) of the wild type and 66.7% (12/18) of the alpha variant were detected by X-rays (p = 0.0246). The main finding of pneumonia was a higher percentage of ground-glass opacities than consolidation in both the wild type and alpha variant. In the alpha variant, multiple signs of air bubbles were observed in four patients on chest CT; however, these were not observed in the wild type (p = 0.014).

Conclusion

The imaging features of pneumonia may be different in variants of COVID-19 compared to those in the wild type. CT helps to detect pneumonia and identify features in patients with COVID-19 because it is difficult to detect COVID-19 pneumonia using plain chest radiographs.

## Introduction

In December 2019, coronavirus disease 2019 (COVID-19) was reported as pneumonia of unknown cause in Wuhan, Hubei Province, China, and infection caused by severe acute respiratory syndrome coronavirus 2 (SARS-CoV-2) [1].

Computed tomography (CT) findings in patients with wild-type SARS-CoV-2 have been reported [2]. For patients with COVID-19, CT is important for the clinical diagnosis and evaluation of disease status. COVID-19 pneumonia typically shows abnormal bilateral shadows in the lung parenchyma, especially ground-glass opacities (GGOs) observed on CT [3]. As the infection progresses, pulmonary consolidation and interstitial changes may become more distinct; however, these findings may vary depending on factors, such as viral strain, patient immune response, age, and underlying disease. Although research on whether there are differences in pulmonary effects and features on CT images between the wild type and alpha variant remains limited, Inui et al. suggested that there is no evidence of any difference in CT findings between the wild-type and alpha variants [4]. Meanwhile, a study has reported that patients infected with the alpha variant may show more extensive lung involvement on CT, which may be associated with more severe diseases [5]. Moreover, mutations in the alpha variant of the spike protein may increase the efficiency of viral entry into cells and cause more severe lung damage [6].

In the present study, patients without respiratory failure were admitted to Fukui General Hospital as inpatients with COVID-19. Chest CT was performed upon admission as part of the index to determine COVID-19 severity. Many young patients are considered to have a mild disease and are rarely examined for pneumonia. However, the presence or absence of pneumonia may alter the severity and treatment of the disease. Here, we report COVID-19 pneumonia in mild cases, including a comparison between the wild type and alpha variant.

## Materials and methods

Study design

This was a retrospective, single-institution study conducted at Fukui General Hospital. This study aimed to evaluate the imaging characteristics of COVID-19 patients diagnosed with either the wild type or alpha variant of SARS-CoV-2. The study was conducted between October 31, 2020, and April 30, 2021, and was designed to analyze chest imaging findings in patients diagnosed with COVID-19 who were admitted to our institution.

Inclusion and exclusion criteria

Patients eligible for inclusion in this study were those diagnosed with COVID-19 based on reverse transcription polymerase chain reaction (RT-PCR) using nasopharyngeal swabs. All enrolled patients were triaged and transported to Fukui General Hospital without oxygen therapy. The inclusion criteria for the study were as follows: adults aged 15 years or older, patients admitted to Fukui General Hospital for COVID-19 treatment, and those who underwent both plain X-rays and CT at their admission. The exclusion criteria were children under the age of 15 years, patients with percutaneous oxygen saturation (SpO2) levels below 94% at admission, and those who did not undergo plain chest radiographs (X-ray) and CT imaging.

Data collection

Data were collected retrospectively from the medical records of patients admitted to Fukui General Hospital. The following patient data were extracted: demographic information, including age, sex, and underlying medical conditions; clinical presentation, such as the number of days from symptom onset to hospital admission; and imaging data, which included X-rays and chest CT scans.

Two experienced radiologists, each with more than 20 years of experience, independently evaluated the chest X-ray and CT images. They were blinded to the patient outcomes, and after conducting independent evaluations, they compared their findings and reached a consensus on the imaging results.

Statistical analysis

Statistical analysis of the comparison between the wild type and alpha variant was performed by the Chi-square test using GraphPad Prism 10 software (GraphPad Software, San Diego, CA, USA). Statistical significance was set at p < 0.05.

Ethics approval

This study was approved by the Research Ethics Committee of our hospital on August 26, 2021 (approval no. 2021-9). The need for informed consent was waived by the Ethics Committee. The authors collected information that could not be used to identify the individual participants.

## Results

Patients’ characteristics

A total of 128 patients with COVID-19 were admitted to our hospital between October 31, 2020, and April 30, 2021. There were 76 and 52 patients with the wild type and alpha variant, respectively (Table 1).

**Table 1:** Patients’ characteristics

Age group	Number of cases	Male: female	Alpha variant, % (n)	Age (mean ± SD)
15-19	25	14: 11	52.0 (13)	16.64 ± 1.15
20-29	41	22: 19	39.0 (16)	23.58 ± 2.76
30-39	17	11: 06	52.9 (9)	34.71 ± 2.54
40-49	22	13: 09	45.5 (10)	45.09 ± 2.31
50-59	14	5: 9	21.4 (3)	52.86 ± 3.23
Over 60s	9	5: 4	11.1 (1)	66.89 ± 5.04
Total	128	70: 58	40.6 (52)	33.65 ± 15.44

**Figure 1:**
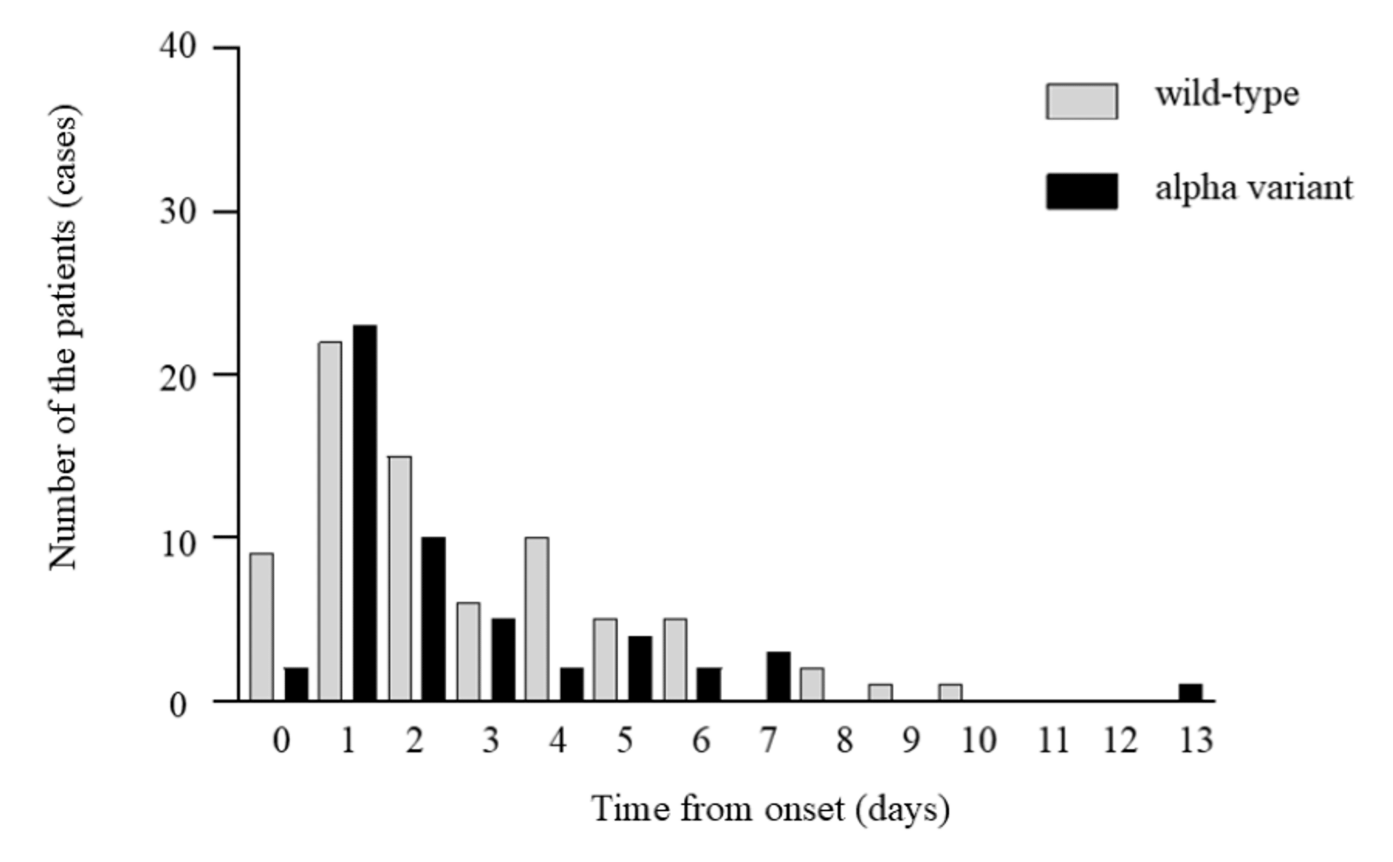
Time from the onset to admission of the patients with wild-type and alpha variant Days from the onset were 5.43 ± 6.62 in patients with wild-type coronavirus disease 2019 (COVID-19) and 3.71 ± 6.19 days in patients with alpha-variant COVID-19. We did not conduct a statistical study between the wild type and the alpha variant.

Days from the onset were 5.43 ± 6.62 and 3.71 ± 6.19 days in patients with wild-type and alpha-variant COVID-19, respectively (Figure 1).

Pneumonia occurrence rate

**Figure 2:**
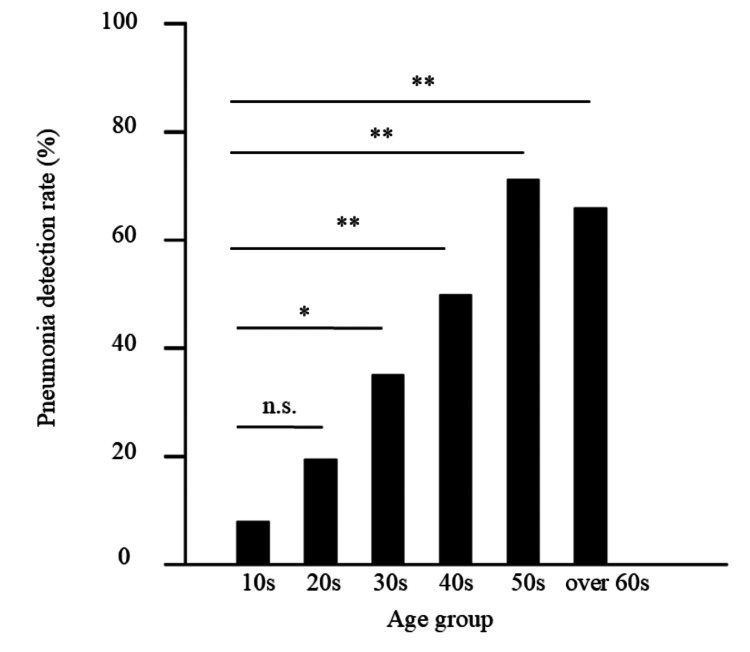
Pneumonia detection rate of all patients The percentage of pneumonia in patients with coronavirus disease 2019 (COVID-19) by the age group was evaluated using computed tomography (CT) (*p < 0.05, **p < 0.01).

The pneumonia occurrence rates obtained using radiography were 10.5% and 23.1% in the wild type and alpha variant, respectively, with no significant difference (p = 0.055). The detection rate using CT was 32.9% for the wild type and 34.6% for the alpha variant (Table 2). Of the pneumonia cases detected by CT, 32.0% (8/25) of wild type and 66.7% (12/18) of the alpha variant were detected by PA X-ray (p = 0.0246). In all patients, the pneumonia detection rate using chest radiography was 15.6% (20/128) and that using CT was 33.6 % (43/128) (p = 0.0008). The pneumonia detection rates in patients in their 30s and 40s were > 30% and > 50%, respectively (Figure 2).

**Table 2:** Imaging findings in the wild type and alpha variant Statistical analysis was performed using the Chi-square test. Statistical significance was set at p < 0.05. GGO: Ground-glass opacity

	Wild type, % (n)	Alpha variant, % (n)	p
Detection rate using X-p	10.5 (8)	23.1 (12)	0.055
Detection rate using CT	32.9 (25)	34.6 (18)	0.85
GGO	13.2 (10)	13.5 (7)	0.96
GGO + Crazy-paving pattern	13.2 (10)	7.7 (4)	0.33
Consolidation	0.07 (5)	13.5 (7)	0.19
Multiple air bubble sign	0.0 (0)	0.08 (4)	0.014

**Figure 3:**
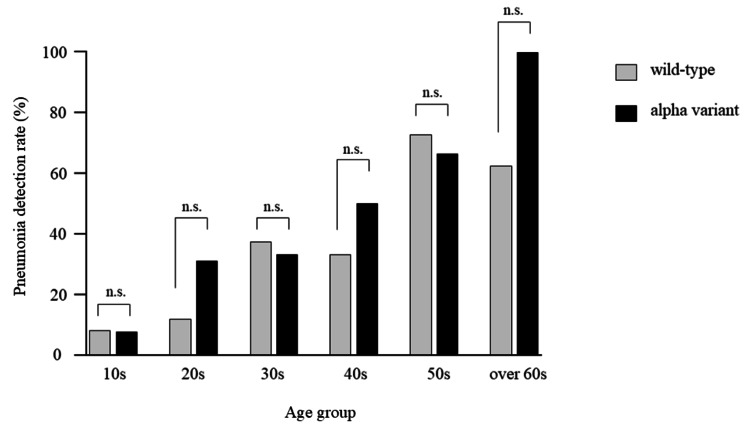
Pneumonia detection rate of the wild type and alpha variant The percentage of pneumonia in patients with COVID-19 by age group in the wild type and alpha variant was evaluated using CT. n.s., not significant.

There was no statistically significant difference in the pneumonia detection rates between the wild type and alpha variant in each age group (Figure 3).

**Figure 4:**
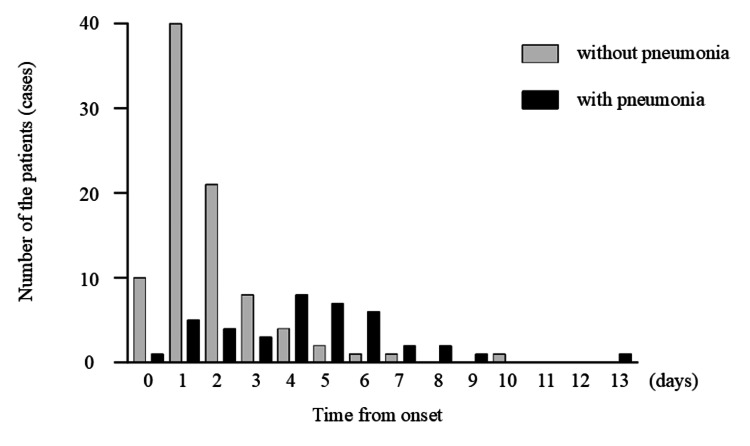
Time from the onset to CT imaging and presence of pneumonia The time since the onset and presence of pneumonia were examined using computed tomography (CT).

Upon admission, 36 patients had been infected for > three days after disease onset, of which 27 (75.0%) had pneumonia, 12 patients (14.1%) had pneumonia out of 92 patients who came to our hospital until three days from the onset (Figure 4).

CT imaging findings

**Figure 5:**
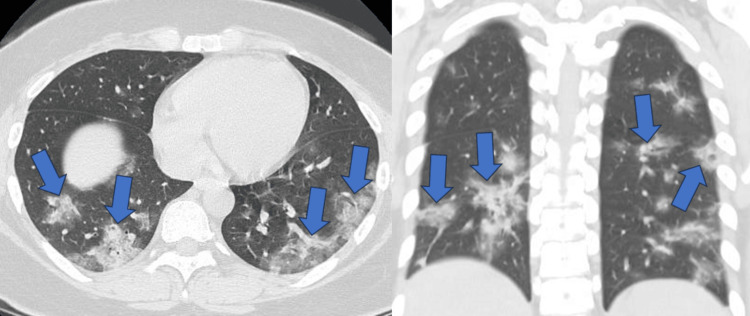
Patchy consolidation in bilateral lungs A female patient in her 20s was admitted to the hospital five days after the onset of symptoms of the alpha variant. Computed tomography (CT) revealed patchy consolidation in both lungs.

**Figure 6:**
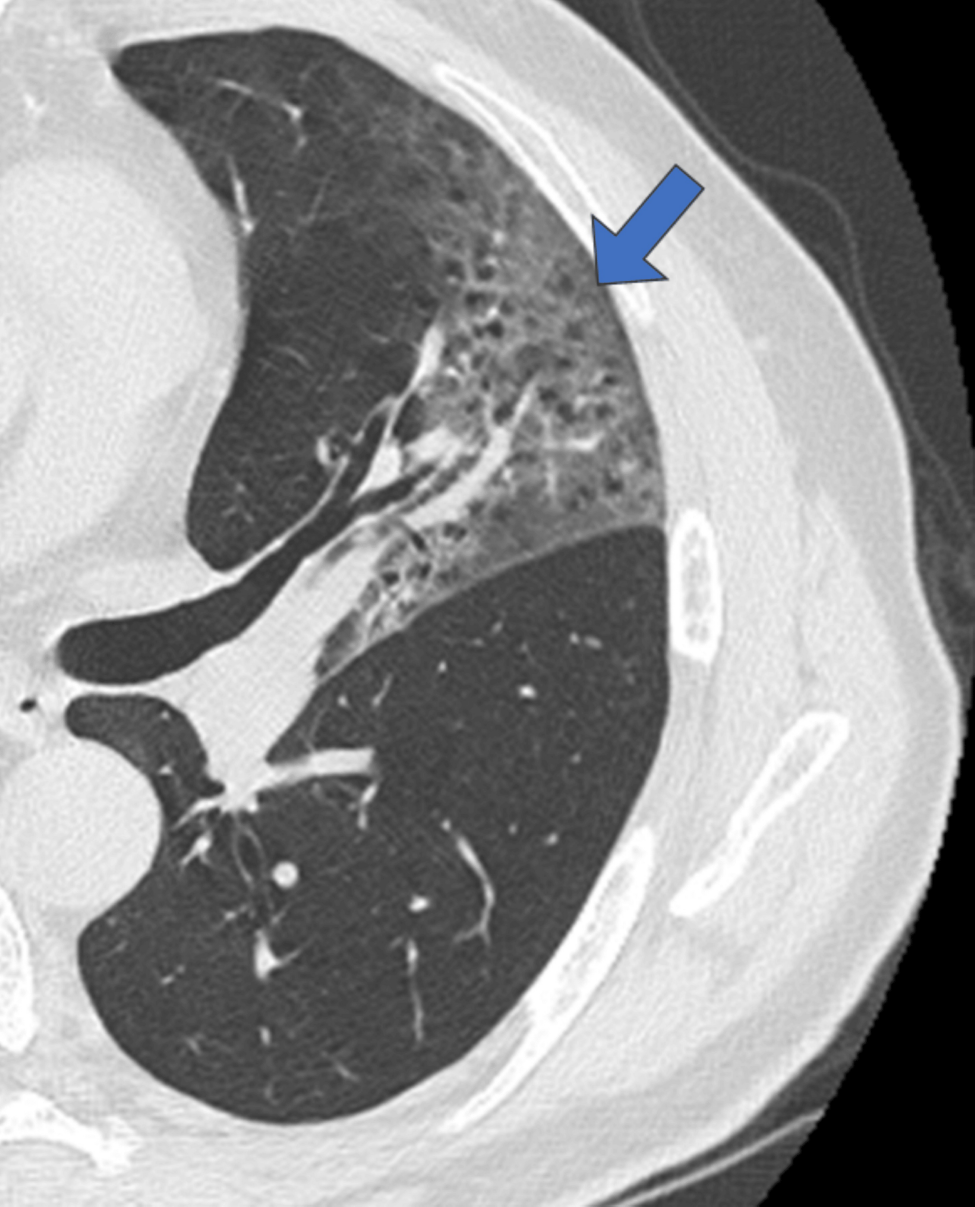
Multiple air bubble signs in ground-glass opacity A man in his 60s with the alpha variant was admitted to the hospital seven days after the disease onset.

Pneumonia findings were classified in CT as GGO, GGO with a crazy-paving appearance, and consolidation. The major findings were GGO, GGO + crazy-paving pattern, and consolidation in 17 (39.5%), 14 (32.6%), and 12 (27.9%) patients, respectively. The main findings were as follows; 10 (40%), 10 (40%), and five (20%) cases of GGO, GGO + crazy-paving pattern, and consolidation in the wild type, and seven (39%), four (22%), and seven (39%) cases of GGO, GGO + crazy-paving pattern, and consolidation in the alpha variant, respectively (Table 2). A representative case of patchy consolidation in bilateral lungs was shown in the alpha variant (Figure 5). Consolidation was more frequent in the alpha variant than in the wild type, with no significant difference (p = 0.304). The alpha variant showed multiple air bubble signs in one case of GGO (Figure 6) and three cases of consolidation; however, no air bubble was observed in the wild type (p = 0.014) (Table 2).

## Discussion

The presence or absence of pneumonia, time since the onset and presence of pneumonia, and findings of pneumonia in patients with COVID-19 were evaluated using chest CT and plain chest radiographs of patients with mild COVID-19 without respiratory failure. There have been reports of patients with COVID-19 pneumonia without respiratory failure, including several reports of asymptomatic cases [7-9]. However, these data were mainly obtained from elderly patients with a mean age of approximately 60 years, and there have been few reports on CT in young patients. In the present study, even in mild COVID-19 cases without respiratory failure, pneumonia was observed in 50% and 71.4% of the patients in their 40s and 50s, respectively. Concerning duration from onset and pneumonia rate, the pneumonia rate was high (75.0%) in cases in which more than three days since the disease onset. The pneumonia rate in patients with moderate disease who did not receive oxygen treatment was lower than that reported by Boeheim et al. [10]. COVID-19 is associated with a high rate of pneumonia as the duration of illness increases. The CT findings of COVID-19 pneumonia, which showed a high rate of GGO with or without a crazy-paving appearance, were consistent with previous reports on COVID-19 pneumonia [8,11].

The detection rate of pneumonia on chest radiography tended to be higher in the alpha variant than in the wild type, and there was a tendency for more shadow. In COVID-19 pneumonia with any variants, the longer the time since onset, the more likely it is that consolidation will occur. The present study revealed that 14.1% of patients with pneumonia came to our hospital within three days from the onset and 75.0% of patients with pneumonia more than three days from the onset. Although the mean time from the onset to CT imaging was shorter for the alpha variant than for the wild type, consolidation was more common in the alpha variant than in the wild type, with no significant difference. The detection rate of pneumonia on plain chest radiographs of pneumonia detected by CT is 66.7%, even in the alpha variant, so CT is necessary to evaluate the presence of pneumonia [12,13].

The present study revealed that multiple air bubble signs were detected only in the alpha variant, and no air bubbles were observed in the wild type (p = 0.014). Ye et al. reported that the air bubble sign indicates expansion of the physiological space in the lesion, bronchiectasis, and consolidation resorption [2]. This finding was considered a round cystic change by Shi et al. [12] and may indicate that the infection may have caused damage to the alveolar wall, resulting in pneumatocele and alveolar damage may be more severe in the alpha variant.

According to the COVID-19 severity classification, severity is classified as moderate I if there are findings of pneumonia, and careful observation after hospitalization is recommended [14]. However, amid the nationwide spread of the infection, pneumonia is hypothesized to be highly prevalent among patients with mild diseases who are isolated at home or treated in overnight care facilities. If possible, chest CT should be performed to classify the severity of the disease, and appropriate treatment should be provided immediately to reduce the rate of severe disease requiring ventilation or extracorporeal membrane oxygenation [15].

We found a high incidence of pneumonia even in young individuals without respiratory failure. In alpha variant cases, there was a high consolidation rate on CT, and some cases showed multiple air bubble signs in a background of dense shadows. The delta and omicron variants were prevalent after outbreak of the alpha variant. Differences in imaging between the delta and omicron variants have already been reported [16], and each variant differs in clinical and imaging characteristics. The typical COVID-19 pneumonia is less common with the omicron variant; however, whether this is due to the weakening of the virulence of the omicron strain or the effect of widespread vaccination is unclear. The present study showed that a certain percentage of patients without respiratory failure had pneumonia with the wild-type and alpha variant that spread during the period when vaccination was not available.

This study has several limitations that should be considered when interpreting the results. First, its retrospective design introduces the potential for selection bias, as the study relied on previously collected data rather than prospectively enrolling participants. Second, the small sample size is another limitation. The relatively limited number of patients included in the study may reduce the statistical power, making it difficult to detect small but potentially meaningful differences between the groups. Finally, the study was conducted at a single institution, which further restricts the generalizability of the findings. Institutional-specific factors, such as the patient population, clinical protocols, and imaging practices, may differ from those in other hospitals or regions, limiting the external validity of the study.

## Conclusions

A high incidence of pneumonia has been observed, even in young individuals without respiratory failure. Only 15.6% (20/128) of the patients were diagnosed with pneumonia using radiography, whereas 33.6% (43/128) of the patients were diagnosed using CT (p = 0.0008). Among the pneumonia cases detected using CT, X-rays detected 32.0% (8/25) in wild-type pneumonia and 66.7% (12/18) in alpha-variant pneumonia (p = 0.0246). The alpha variant showed that multiple air bubble signs were detected using CT only in the alpha variant, and no air bubbles were observed in the wild type (p = 0.014). CT is extremely useful for detecting COVID-19 pneumonia and identifying the features of the variant. Moreover, the treatment differs depending on the presence of pneumonia, and an appropriate diagnosis using CT imaging is necessary.

## References

[1] Huang C, Wang Y, Li X (2020). Clinical features of patients infected with 2019 novel coronavirus in Wuhan, China. Lancet.

[2] Ye Z, Zhang Y, Wang Y, Huang Z, Song B (2020). Chest CT manifestations of new coronavirus disease 2019 (COVID-19): a pictorial review. Eur Radiol.

[3] Kwee TC, Kwee RM (2020). Chest CT in COVID-19: what the radiologist needs to know. Radiographics.

[4] Inui S, Fujikawa A, Gonoi W (2022). Comparison of CT findings of coronavirus disease 2019 (COVID-19) pneumonia caused by different major variants. Jpn J Radiol.

[5] Simon J, Grodecki K, Cadet S (2022). Radiomorphological signs and clinical severity of SARS-CoV-2 lineage B.1.1.7. BJR Open.

[6] Davies NG, Abbott S, Barnard RC (2021). Estimated transmissibility and impact of SARS-CoV-2 lineage B.1.1.7 in England. Science.

[7] Goldenfeld M, Nir-Paz R, Segal G (2021). Characteristics of clinically asymptomatic patients with SAES-CoV-2 infections, case series. Prehosp Disaster Med.

[8] Inui S, Fujikawa A, Jitsu M (2020). Chest CT findings in cases from the cruise ship “Diamond Princess” with coronavirus disease 2019. Radiol Cardiothorac Imaging.

[9] Bandirali M, Sconfienza LM, Serra R, Brembilla R, Albano D, Pregliasco FE, Messina C (2020). Chest radiograph findings in asymptomatic and minimally symptomatic quarantined patients in Codogno, Italy during COVID-19 pandemic. Radiology.

[10] Bernheim A, Mei X, Huang M (2020). Chest CT findings in coronavirus disease- 2019(COVID-19): relationship to duration of infection. Radiology.

[11] Zhao W, Zhong Z, Xie X, Yu Q, Liu J (2020). Relation between chest CT findings and clinical conditions of coronavirus disease (COVID-19) pneumonia: a multicenter study. AJR Am J Roentgenol.

[12] Shi H, Han X, Jiang N (2020). Radiological findings from 81 patients with COVID-19 pneumonia in Wuhan, China: a descriptive study. Lancet Infect Dis.

[13] Zu ZY, Jiang MD, Xu PP, Chen W, Ni QQ, Lu GM, Zhang LJ (2020). Coronavirus disease 2019 (COVID-19): a perspective from China. Radiology.

[14] Wang Y, Dong C, Hu Y (2020). Temporal changes of CT findings in 90 patients with COVID-19 pneumonia: a longitudinal study. Radiology.

[15] Horby P, Lim WS, Emberson JR (2021). Dexamethasone in hospitalized patients with Covid-19. N Engl J Med.

[16] Tsakok MT, Watson RA, Saujani SJ (2023). Reduction in Chest CT severity and improved hospital outcomes in SARS-CoV-2 Omicron compared with delta variant infection. Radiology.

